# Audiometric Findings in Senior Adults of 80 Years and Older

**DOI:** 10.3389/fpsyg.2022.861555

**Published:** 2022-07-20

**Authors:** Kaat De Raedemaeker, Ina Foulon, Roberta Vella Azzopardi, Elke Lichtert, Ronald Buyl, Vedat Topsakal, Ingo Beyer, Ivan Bautmans, Olaf Michel, Frans Gordts

**Affiliations:** ^1^Department of Otorhinolaryngology Head and Neck Surgery, Universitair Ziekenhuis Brussel, Vrije Universiteit Brussel, Brussels, Belgium; ^2^Gerontology Department and Frailty in Aging Research (FRIA) Group, Faculty of Medicine and Pharmacy, Vrije Universiteit Brussel, Brussels, Belgium; ^3^Department of Public Health, Biostatistics and Medical Informatics Research Group, Vrije Universiteit Brussel, Brussels, Belgium; ^4^Department of Geriatrics, Universitair Ziekenhuis Brussel, Brussels, Belgium

**Keywords:** presbycusis or age-related hearing impairment (ARHI), hearing aids (HA), sex, speech audiometry, pure-tone audiometry (PTA)

## Abstract

**Objective:**

To examine hearing thresholds in senior adults of 80 years and older and compare this data to the current ISO 7029 reference values.

**Design:**

A descriptive, prospective study testing pure-tone and speech audiometry in senior adults participating in the BUTTERFLY study or the BrUssels sTudy on The Early pRedictors of FraiLtY. A Gerontological study to identify determinants for active aging and for early stages of frailty in the oldest population. Using the formula given by ISO 7028:2017 the median value of hearing was calculated based on the sex and age of the participant and compared to the measured hearing thresholds.

**Results:**

151 senior adults were included. The prevalence of hearing loss was 90.7% (PTA > 20 dB HL). The results were compared to the mean ISO values, calculated for every participant. Both males and females in our study population had worse hearing thresholds than could be expected based on the ISO reference values. In our study population with moderate hearing loss (PTA > 40 dB HL), 38% is underserved in term of hearing restoration healthcare and yet another 38% is unsatisfied with the result of the hearing aids. Given the vast impact on the individual and society, this is a problem in need of our attention.

**Conclusion:**

The ISO 7029 reference values may be an underestimation of hearing loss in senior adults of 80 years and older. Therefore we present a statistical distribution of hearing thresholds on different frequencies related to age and sex that can be used as a baseline for further development of the reference values.

## Introduction

According to the latest Global Burden of Disease Studies (GBD 2019 Diseases and Injuries Collaborators, 2020), hearing loss is one of the six most important drivers of increasing burden (disability-adjusted life-years) in older adults. In 2015 hearing loss was one of the eight leading causes of chronic disease and injury. It affected more than 10% of the world’s population and was the second greatest impairment based on the number of individuals affected (1.33 billion). In over 90% of these cases the cause of the hearing loss was classified as Age-Related Hearing Loss (ARHL). ARHL, also known as presbycusis, is a term used to define a subtle and progressive bilateral hearing loss with a sensorineural origin related to increasing age. It is known that higher age is associated with higher prevalence of hearing loss. Considering that the global population is aging, it is important that we recognize the burden of hearing loss in this group. The most vulnerable frequencies in the hearing of this age category are above 1,000 Hz. However, research suggests a clear difference between males and females (Weinstein, 2002). Males mostly suffer a moderate to severe hearing loss and deteriorate more in the high frequencies which is clearly illustrated by the sharply sloping pattern of their pure-tone audiogram. Females mostly suffer a mild to moderate hearing loss and also deteriorate in the low frequencies which gives them a more gradually sloping curve.

Within older adults, hearing loss has an important impact on the individual. It is not only associated with social isolation (Mick et al., 2014), but also with functional decline (Gopinath et al., 2012), depression (Kramer et al., 2002), adverse effects on quality of life (Dalton et al., 2003), dementia (Lin et al., 2011a; Deal et al., 2017) and cognition (Taljaard et al., 2016). Recent studies show that hearing loss is one of the largest potentially modifiable factors to reduce the risk of dementia (Livingston et al., 2020). The use of hearing aids could therefore be a major protective factor for people at risk of cognitive decline and dementia (Maharani et al., 2018). Unfortunately, a large group of older adults with hearing loss remain untreated or even undiagnosed (Hartley et al., 2010). Screening for hearing loss in a senior population could therefore have benefits not only for hearing but for general health as well (Gates and Mills, 2005).

The third and most recent edition of the International Organization for Standardization (ISO) standards for the statistical distribution of hearing thresholds related to age and sex addresses hearing thresholds for adults from 18 up to the age of, 80 years (Technical Committee ISO/TC 43 Acoustics, 2017), who have no history of noise exposure, cerumen impaction or ear pathologies. Unfortunately, there are no such standards for the population above 80 years. Since the BUTTERFLY gerontological study, conducted at the Vrije Universiteit Brussel, does include healthy senior adults of 80 years and older, the opportunity was seized to provide additional data that eventually could be of interest for the ISO technical committees for preparing future International Standards. These Belgian data could complement those that have been provided in the past.

A better knowledge of the hearing thresholds among the most elderly is not only of importance to the subjects themselves, but also to clinicians and researchers. For the patients, their families and the clinicians, it can be important to be aware whether or not an individual subject presents a hearing threshold that can be expected for that age, and if more than the usual supportive measures have to be provided.

In this study we will determine hearing thresholds related to age and sex in otologically normal senior adults of 80 years and older in a Belgian population and compare them to the ISO hearing thresholds calculated for the same age category by using the formula given by ISO 7028:2017.

## Materials and Methods

The study protocol was approved by “the Committee of Medical Ethics” of the Universitair Ziekenhuis Brussel. It is an explorative, prospective, observational study that started in September 2015 with a longitudinal follow up over 2 years. This study is part of a larger gerontological study that examines various aspects of the health of senior adults over 80 years of age, including Ear, Nose and Throat (ENT) tests. The study is called the BUTTERFLY or BrUssels sTudy on The Early pRedictors of FraiLtY. The aim is to identify determinants for active aging (assets) and for early stages of frailty (deficits in reserve capacity) in the oldest population.

### Subjects

All participants were senior adults who were over 80 years old or who turned 80 in the year of participation. They were community dwelling and well-functioning adults recruited through advertisements at the Universitair Ziekenhuis Brussel, but also in elderly care organizations, health insurance companies, general practitioners and pharmacies. Inclusion criteria were: walking independently and living at home independently. All participants underwent medical and physical testing as well as psychosocial and cognitive testing. A thorough ENT examination and audiometric analysis were part of the medical testing in this study.

Exclusion criteria were an aberrant tympanometry type (B, C and D), ear pathology with known impact on hearing threshold (Menière’s disease, vestibular schwannoma, e.g.) ear surgery and chronic ear pathology (ear drum perforation, chronic ottorhea, e.g.).

Given the unreliable and unstandardized information concerning noise exposure, familial history of SNHL and the use of ototoxic medication, these were not included in the exclusion criteria.

During the study period from September 2015 to September 2016, a total of *N* = 181 participants, 95 males and 86 females, were interviewed and examined at the ENT Department. One hundred fifty-one participants met the inclusion criteria. Of the 30 participants who were excluded from the study, 17 had an aberrant tympanogram, 9 had a disease that is known to cause hearing loss, 4 had chronic infections and perforation of the eardrum and 4 had a history of ear surgery. There were 3 participants with more than one exclusion criterium. The diseases associated with hearing loss were very diverse: otosclerosis (*n* = 2), unilateral self-reported deafness (*n* = 2), Menière’s disease (*n* = 1), cholesteatoma (*n* = 1), vestibular schwannoma (*n* = 1) and sequelae of sudden deafness (*n* = 1). There were only 2 types of ear surgery: attico-antro-mastoidectomy (*n* = 2) and ossicular chain reconstruction (*n* = 2).

Of the 151 included participants 84 (55.6%) were male and 67 (44.4%) were female. The mean age was 82.4 years old (SD ± 2.54), with a minimum age of 80 and a maximum age of 90. One hundred twenty-one (80%) participants belonged to age group 1 of which 70 (57.9%) male and 51 (42.1%) female. Thirty (20%) belonged to age group 2 of which 14 (46.7%) male and 16 female (53.3%). There were 31 hearing aid users (20.5%) and among them 12 (38.7%) were unsatisfied with the effect of their amplification. Nine (6%) participants spontaneously stated to have regular tinnitus complaints. Forty five (29.8%) participants stated that they had been exposed to noise, the majority was male (*n* = 37) of which 32 cases had a history of professional noise exposure mostly due to heavy machinery (*n* = 17) and police- and military work (*n* = 15). Among the 8 females with self-reported noise exposure, all the exposure was work related and mostly due to teaching and professional phone usage. The history of ototoxic medication use was reported in 6 participants (4%) (2 cases in the context of chemotherapy for cancer treatment and 4 cases of Quinine use). A familial history of hearing loss, whether or not with an early onset, was reported in 24 (15.9%) of the participants (Table 1).

**TABLE 1:** Demographic information of all participants and results of the standardized questionnaire.

	Male	Female	Total
Age group 1	70	51	121
Age group 2	14	16	30
Hearing aid use	18	13	31
Tinnitus	5	4	9
Noise exposure	37	8	45
Ototoxic medication	4	2	6
Familial history of hearing loss	7	17	24

### Audiometric Assessment

All participants underwent a basic ENT examination and completed a standardized questionnaire with an ENT specialist at the Universitair Ziekenhuis Brussel. The following risk factors for hearing loss were recorded: self-reported ear pathologies, familial history of hearing loss, noise exposure, use of ototoxic medication and history of ear surgery. The use and benefit of hearing aids was reported.

The audiometric assessments consisted of tympanometry, pure-tone and speech audiometry and were performed without the use of hearing aids. All audiometric tests were performed by experienced audiometricians of the Universitair Ziekenhuis Brussel.

Tympanometry was performed by using an impedance audiometer (MADSEN OTOflex 100: Veranneman, 1000 Brussel, België) with a 226 Hz probe tone. The tympanometry graph was subcategorized by using the Liden and Jerger classification system (Liden, 1969; Jerger, 1970). Tympanometry type A (normal tympanogram with a peak within the reference range), As (hypo mobile eardrum with admittance < 0.30 mmho) and Ad (hyper mobile eardrum with admittance > 1.60 mmho) were considered to reflect a normal aerated ear space and used as inclusion criteria. Tympanometry type B (flat tympanogram), C (negative pressure < −100 daPa) and D (M-shaped tympanogram) were considered pathological and excluded from this study.

Pure-tone audiometry included the determination of the air- and bone conduction hearing thresholds with a liminar tonal audiometric (LTA) examination using the International Organization for Standardization: ISO 8253-1:2010 methods (Technical Committee ISO/TC 43 Acoustics, 2010) for pure-tone audiometry and masking. More specifically, we used the Hughson-Westlake method with the masking technique by Hook. Tests were executed in a high quality audiometry sound attenuated room (Veranneman, 1000 Brussel, België) using a MADSEN Astera2 clinical audiometer (Veranneman, 1000 Brussel, België) with a TDH39 headphone and B-71 bone oscillator. All tests were conducted with the same material which was timely calibrated by a professional. Air-conduction thresholds were measured at frequencies 125, 250, 500, 750, 1,000, 1,500, 2,000, 3,000, 4,000, 6,000, and 8,000 Hz. Bone-conduction was measured at the frequencies 250, 500, 750, 1,000, 1,500, 2,000, 3,000, and 4,000 Hz. The pure-tone average (PTA) (500, 1,000, 2,000, and 4,000 Hz) was chosen to quantify the degree of sensorineural hearing loss (SNHL) and to determine the better ear per subject according to the classification of the Bureau International d’ Audiophonologie (BIAP) [Bureau International d’ Audiophonologie [BIAP], 1997]. To analyze the pure-tone audiometry according to various frequency intervals, different averages were calculated: The low frequency average (125, 250 and 500 Hz), the pure-tone average (PTA) (500, 1,000, 2,000 and 4,000 Hz) and the high frequency average (4,000, 6,000 and 8,000 Hz) (Hannula et al., 2010). Contrary to the “low and high” Fletcher averages, often used in everyday clinical practice, these three frequency averages have been used to take a more detailed look at hearing thresholds. All frequencies needed to be evaluated to describe the differences between high and low frequencies and the effect of sex and age. This is why apart from the PTA 0.5–4 recommended by EU expert group, we calculated the high and low frequency averages. Especially the high frequency average is of great interest, as presbycusis is mostly a high frequency problem. The air-conduction thresholds were tested for asymmetry between the right and left ear using the definition according to BIAP where a unilateral or asymmetric hearing loss is defined as an air-conduction PTA difference of 15 dB or greater.

For the classification of hearing loss, the BIAP classification was used, as it is a well know and frequently used classification in Belgium and Europe, and is largely comparable to the grades of hearing impairment recommended by the Global Burden of Disease Expert Group on Hearing Loss (Olusanya et al., 2019) and the ASHA classification (Clark, 1981).

During speech audiometry the right and left ear were tested separately. According to the native language of the subject, Dutch or French standardized two-syllable word lists were used. The native Dutch speaking group was tested with the standardized BLU-wordlist (Wouters et al., 1994) and the native French speaking group with the Fournier wordlist (Fournier, 2006). These two types of word lists in Dutch and French both consist of two syllable words with a comparable norm curve. The speech reception threshold (SRT) was automatically calculated from the speech audiogram as the lowest level at which a speech signal is understandable enough to be recognized and repeated 50% of the time. Other parameters recorded were: whether or not the 100% speech recognition was accomplished, at what sound level (in dBSPL) it was accomplished and the presence of a roll-over. Roll-over was defined as a diminishing speech discrimination seen with increasing sound intensity after reaching a maximal Performance-Intensity score.

### Comparison of BUTTERFLY Data to ISO Standard

To make a comparison between BUTTERFLY data and ISO standard statistical distribution of hearing thresholds related to age and sex, we used the formula given by ISO 7028:2017 to calculate the median value of hearing for males and females on different frequencies for different ages: ΔH_md,Y_ = α_md_ (Y – 18)^β^ *^md^*. “Y” is age in years, α_md_ and β_md_ are dimensionless quantities given by ISO for males and females for frequencies from 125 to 8,000 Hz (Table 2). The standard median value based on their sex and age was calculated for every participant for frequencies from 125 Hz to 8,000 Hz. A comparison was made between the mean measured hearing thresholds and the calculated ISO thresholds for every frequency for males and females. By using the area under the curve, where all frequencies were equally important, a general comparison was made between the measured thresholds and the calculated ISO thresholds. If the true value of the frequencies were used, the higher frequencies will have a relative higher influence than the lower frequencies in this calculation (i.e., the difference between 125 and 250 Hz is much smaller than the difference between 4,000 and 8,000 Hz). This wouldn’t be representative for the overall audiogram.

**TABLE 2:** Values of α_md_ and βmd given By ISO 7029/2017 (Technical Committee ISO/TC 43 Acoustics, 2017).

Frequency	α_md_		β_md_	
	
Hz	Males	Females	Males	Females
125 250 500 750 1,000 1,500 2,000 3,000 4,000 6,000 8,000	2.50 × 10^–6^ 1.39 × 10^–4^ 4.59 × 10^–4^ 5.70 × 10^–4^ 7.02 × 10^–4^ 1.09 × 10^–3^ 1.56 × 10^–3^ 2.54 × 10^–3^ 3.40 × 10^–3^ 4.53 × 10^–3^ 5.06 × 10^–3^	6.16 × 10^–4^ 3.98 × 10^–4^ 2.61 × 10^–4^ 2.25 × 10^–4^ 2.21 × 10^–4^ 2.53 × 10^–4^ 3.12 × 10^–4^ 4.88 × 10^–4^ 7.37 × 10^–4^ 1.47 × 10^–3^ 2.53 × 10^–3^	3,841 2,832 2,537 2,512 2,494 2,446 2,404 2,350 2,325 2,315 2,328	2,451 2,568 2,708 2,775 2,805 2,813 2,792 2,728 2,660 2,539 2,439

### Categories

To evaluate confounding factors, participants were grouped into different subgroups according to sex and age: the youngest group or ‘Age group 1’ contained participants from 80 to 84 years old and the oldest group or ‘Age group 2’ those of 85 years and older.

The evaluation for confounding factors was done using the values of the better hearing ear. The pure-tone values were grouped by different frequencies using the low frequency average, PTA and the high frequency average.

### Statistical Analyses

The statistical analysis was carried out using SPSS Statistics computer package, version 23. Descriptive analysis were given by frequencies and percentages for categorical data, mean (standard deviations) for continuous data. When comparing the audiometric data, the Independent sample- or Paired samples Student’s *t*-test was used in the cases with two independent or pairwise variables. To analyze the effect of sex and age groups, and their interaction, on the audiometric data a two-way ANOVA model was used. To verify the homogeneity of variances across independent groups, the Levine’s test was used. To analyze the distribution of the data, the Chi Square test and the Fisher exact test were used. We tested the correlation using the Interclass correlation coefficient. Finally, the area under the curve (AUC) was calculated to compare the values of our measurements and the calculated ISO values using an independent *t*-test. All statistical analyses were performed using a level of statistical significance of 0.05.

## Results

### Pure-Tone Audiometry

**FIGURE 1:**
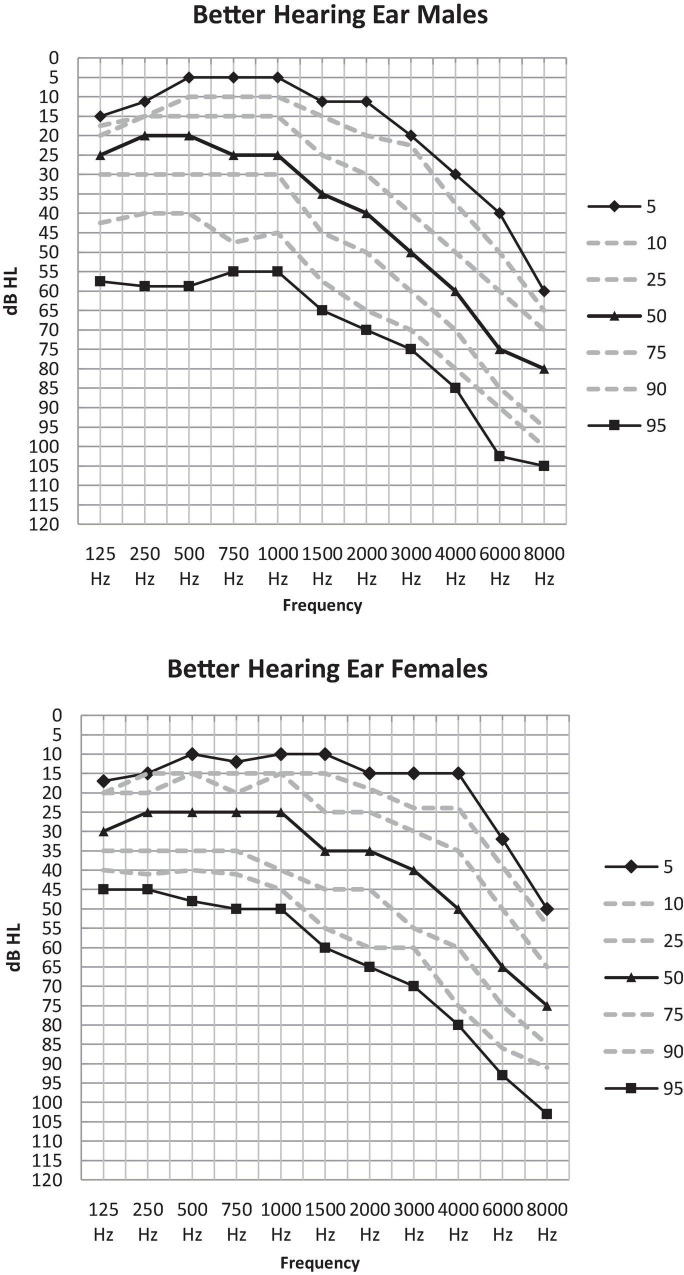
Percentile distribution of the air-conduction thresholds (dB HL) in the better hearing ear in males and females separately.

Table 3A illustrates the percentile distribution of the air-conduction hearing thresholds of our sample, stratified by sex and age. In Table 3B the mean (+ standard deviation) air-conduction thresholds (dBHL) are added for more detail. In Figure 1 we can see that the percentile curves of both males and females are sloping downward in the high frequencies.

**TABLE 3A:** Reference values of hearing thresholds in senior adults of 80 years and older: percentile distribution of the air-conduction thresholds (dB HL) in the better hearing ear in males and females.

Percentiles		125 Hz	250 Hz	500 Hz	750 Hz	1,000 Hz	1,500 Hz	2,000 Hz	3,000 Hz	4,000 Hz	6,000 Hz	8,000 Hz
Male	5	15.00	11.25	5.00	5.00	5.00	11.25	11.25	20.00	30.00	40.00	60.00
	10	17.50	15.00	10.00	10.00	10.00	15.00	20.00	22.50	37.50	50.00	65.00
	25	20.00	15.00	15.00	15.00	15.00	25.00	30.00	40.00	50.00	60.00	70.00
	**50**	**25.00**	**20.00**	**20.00**	**25.00**	**25.00**	**35.00**	**40.00**	**50.00**	**60.00**	**75.00**	**80.00**
	75	30.00	30.00	30.00	30.00	30.00	45.00	50.00	60.00	70.00	85.00	95.00
	90	42.50	40.00	40.00	47.50	45.00	57.50	65.00	70.00	80.00	90.00	100.00
	95	57.50	58.75	58.75	55.00	55.00	65.00	70.00	75.00	85.00	102.50	105.00
Female	5	17.00	15.00	10.00	12.00	10.00	10.00	15.00	15.00	15.00	32.00	50.00
	10	20.00	15.00	15.00	15.00	15.00	15.00	19.00	24.00	24.00	39.00	54.00
	25	20.00	20.00	15.00	20.00	15.00	25.00	25.00	30.00	35.00	50.00	65.00
	**50**	**30.00**	**25.00**	**25.00**	**25.00**	**25.00**	**35.00**	**35.00**	**40.00**	**50.00**	**65.00**	**75.00**
	75	35.00	35.00	35.00	35.00	40.00	45.00	45.00	55.00	60.00	75.00	85.00
	90	40.00	41.00	40.00	41.00	45.00	55.00	60.00	60.00	75.00	86.00	91.00
	95	45.00	45.00	48.00	50.00	50.00	60.00	65.00	70.00	80.00	93.00	103.00

*Bold values represent the P50 or median, which is the most important value in the table.*

**TABLE 3B:** Mean (+ standard deviation) air-conduction thresholds (dB HL) in the better hearing ear in males and females.

			125 Hz	250 Hz	500 Hz	750 Hz	1,000 Hz	1,500 Hz	2,000 Hz	3,000 Hz	4,000 Hz	6,000 Hz	8,000 Hz
Male	Mean		27.62	25.54	23.15	25.00	25.65	35.65	39.64	49.40	60.24	72.14	81.49
	SD (±)		11.21	12.64	13.68	13.58	14.01	16.35	16.93	17.14	16.84	16.60	14.97
Female	Mean		28.81	27.61	27.09	27.69	27.99	34.78	37.16	41.19	48.06	63.51	75.75
		SD (±)	8.49	8.98	10.98	11.26	12.73	15.56	15.60	15.98	18.42	18.01	14.96

#### Classification of Hearing Loss

In the better hearing ear the overall prevalence of hearing loss according to the BIAP classification (PTA > 20 dB HL) was 90.7%, this is 95.2% of all males and 85.1% of all females (Table 4). Most of the participants (57.6%) were situated in the mild hearing loss group and 31.8% suffered from moderate hearing loss. Only 1.3% (*n* = 2) had a 1st degree severe hearing loss. In total 50 (33.1%) of our participants had a hearing loss > 40 dB.

**TABLE 4:** The BIAP classification of hearing impairment in the best- and worse hearing ear.

				Best Ear		
				Male	Female	Total
I. Normal or subnormal hearing		<20 dB	N	4	10	14
			**%**	**4.8**	**14.9**	**9.3**
II. Mild hearing loss		21–40 dB	N	51	36	87
			**%**	**60.7**	**53.7**	**57.6**
III. Moderate hearing loss	1st degree	41–55 dB 56–70 dB	N	23	19	42
	2nd degree		% N	**27.4**	**28.4**	**27.8**
				4	2	6
			**%**	**4.8**	**3.0**	**4.0**
IV. Severe hearing loss	1st degree	71–80 dB	N	2	0	2
			**%**	**2.4**	**0.0**	**1.3**
	2nd degree	81–90 dB	N	0	0	0
			**%**	**0.0**	**0.0**	**0.0**

Bold values represent the percentages of participants for that specific hearing classification, first of all males, then of all females and then of the total population. For example: 4.8% of our male population had a normal hearing.

#### Different Hearing Threshold Averages According to Main Variables: Age and Sex

**FIGURE 2:**
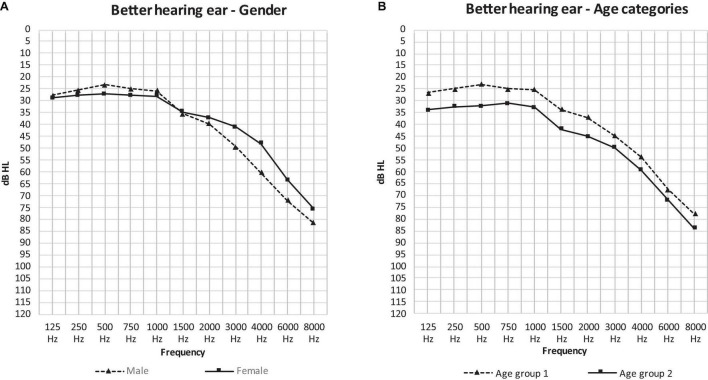
The mean air-conduction thresholds (dB HL) in the better hearing ear of all participants according to gender **(A)** and age group **(B)**.

When comparing the curves of the mean air-conduction thresholds according to sex in Figure 2A, we saw no significant difference in the low frequencies in males compared to females (25.44 and 27.84 dB HL: *p* = 0.7). On the other hand, males had significantly poorer hearing than females according to the PTA (37.17 and 35.07 dB HL: *p* = 0.016) and the high frequency average (71.29 and 62.44 dB HL: *p* < 0.001). This difference can be observed in Figure 2A, where the curve of the males has a steeper slope than that of the females as it crosses the curve of females at 1,500 Hz.

Figure 2B presents the curves of the mean air-conduction thresholds subdivided into two age categories. Hearing loss was worse in age group 2 at every frequency. Age group 1 had significantly better hearing than age group 2 in all threshold averages: the low frequency average (24.93 and 32.83 dB HL: *p* < 0.001), the PTA (34.74 and 42.29 dB HL: *p* = 0.001) and the high frequency average (66.28 and 71.72 dB HL: *p* = 0.031).

The analysis above needs to be interpreted with caution, as the effect of age and sex on the hearing thresholds have a significant interaction (PTA: *p* = 0.027, LFA: *p* = 0.031, HFA: *p* = 0.119).

**FIGURE 3:**
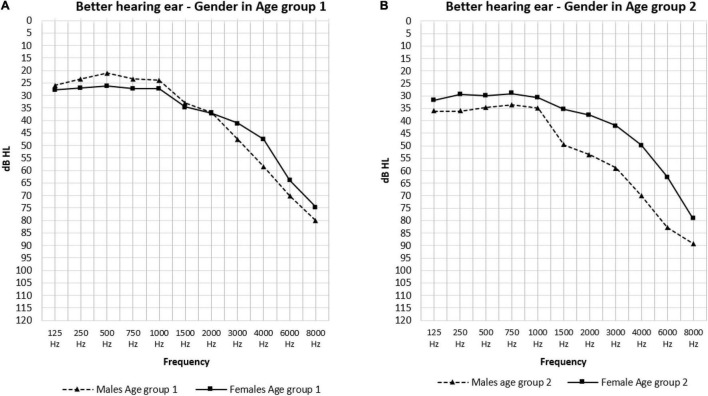
The mean air-conduction thresholds (dB HL) in the better hearing ear according to gender for Age group 1 **(A)** and 2 **(B)** separately.

**FIGURE 4:**
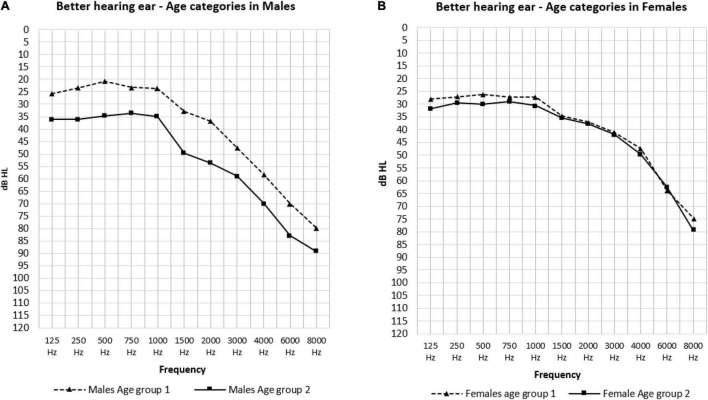
The mean air-conduction thresholds (dB HL) in the better hearing ear according to age groups for males **(A)** and females **(B)**.

The effect of sex is shown in Figure 3. In the low frequencies, males in age group 1 had significantly better hearing thresholds than females (23.40 and 27.03 dB HL: *p* = 0.021) (Figure 3A). Conversely, in the PTA frequencies we saw significantly better hearing thresholds in females in age group 2 than males in this same age group (37.03 and 48.30 dB HL: *p* = 0.026) (Figure 3B). The effect of age was only present in males as can be observed in Figure 4 where the hearing thresholds of the males in age group 1 were significantly better than in age group 2 (PTA: 34.95 and 48.30 dB HL, LFA: 23.40 and 35.59 dB HL, HFA: 69.40 and 80.71 dB HL *p* < 0.001) (Figure 4A). This difference was not present in the hearing thresholds of females (PTA: 34.46 and 37.03 dB HL: *p* = 0.469, LFA: 27.03 and 30.42 dB HL: *p* = 0.156, HFA: 61.99 and 63.85 dB HL: *p* = 0.119) (Figure 4B).

#### The Effect of Hearing Aids

Hearing aid users (*n* = 31) had significantly poorer air-conduction thresholds than subjects without hearing aids (*n* = 120). This difference was present in the PTA frequencies (50.48 and 32.56 dB HL: *p* < 0.001) as well as in the low (33.44 and 24.71 dB HL: *p* = 0.003) and high frequencies (79.30 and 64.28 dB HL: *p* < 0.001). There was no significant difference in the distribution of males and females in the hearing aid user group (Male *n* = 18, Female *n* = 13: *p* = 0.461). Conversely, when analyzing the distribution in age groups we saw that there were significantly more hearing aid users in age group 2: 43.33% vs. 14.88% in age group 1 (*p* = 0.01).

In total 50 (33.1%) of our participants had a hearing loss > 40 dB: these participants could all benefit from wearing hearing aids, but only 31 (62%) of them wore them. This means 19 (38%) participants with moderate hearing loss or more were undertreated by not wearing hearing aids. Moreover, when evaluating the satisfaction of hearing aid users to determine whether they benefited from their hearing aid, there were 12 (38.7%) participants unsatisfied by the effect. Therefore 38.7% of the participants with moderate hearing loss or more who wore hearing aids were undertreated as well.

#### The Effect of Tinnitus

There were 9 participants who spontaneously complained about regular tinnitus symptoms. We couldn’t detect a significant difference in the PTA of participants who did or did not complain about tinnitus (30.97 and 36.37 dB HL: *p* = 0.188). nor did we find a significant difference in the low-(22.41 and 26.76 dB HL: *p* = 0.227) or high frequency averages (64.07 and 67.57 dB HL: *p* = 0.515) in these participants. There were no significant differences in sex (Male: *n* = 5, Female *n* = 4: *p* = 1.00) in the prevalence of tinnitus according to the Fisher’s exact test.

### Speech Audiometry

#### Speech Reception Threshold

There were no significant differences between the mean SRT of the right and left ear (39.57 and 40.55 dBSPL: *p* = 0.379) (Table 5). Therefore, further analyses used the values of the better hearing ear. An interaction between age and sex was observed (*p* = 0.01). Therefore, the groups were analyzed in detail. In age group 2, the SRT of males was significantly poorer than that of females (49.08 and 38.50 Dbspl: *p* = 0.022). In age group 1, sex had no significant effect on the SRT value (36.13 and 37.76 dBSP: *p* = 0.429). In males, we saw that age group 2 had a significantly poorer SRT than age group 1 (49.08 and 36.13 dBSPL: *p* = 0.001). Females showed no significant difference according to age groups (37.76 and 38.50 dBSPL: *p* = 0.796). Hearing aid users had a significantly poorer SRT than subjects without hearing aids (49.47 and 35.21 dBSPL: *p* < 0.001).

**TABLE 5:** Speech reception threshold (dB SPL) according to main variables age and sex.

		N	Mean SRT (dB SPL)	SD (±)	Median SRT (dB SPL)
Age group 1	Total	121	36.82	11.19	35.00
	Male	70	36.13	11.89	34.50
	Female	51	37.76	10.18	36.00
Age group 2	Total	30	43.24	12.61	44.00
	Male	14	49.08	14.36	45.00
	Female	16	38.50	8.84	36.50

#### 100% Speech Recognition

Of all participants, 92.72% reached 100% speech recognition in each ear. In 11 (7.28%) participants, speech recognition scores did not reach 100% in one (*n* = 7) or both (*n* = 4) ears. Only in age group 2 there was a difference in sex where males needed a significantly higher dB level than females (70.00 and 56.00 dB HL: *p* = 0.01) to reach 100%.

#### Presence of Roll-Over

Ninety-one participants showed a roll-over in speech audiometry, 70 in age group 1 (57.85%) and 21 in age group 2 (70.00%). This difference according to age groups was not significant (*p* = 0.298). In total 61.90% of the males and 58.20% of the females showed a roll-over. This difference in sex was not significant (*p* = 0.738). We can conclude that there was no significant effect of age and sex on the prevalence of a roll-over.

### Comparison of Pure-Tone and Speech Audiometry

**FIGURE 5:**
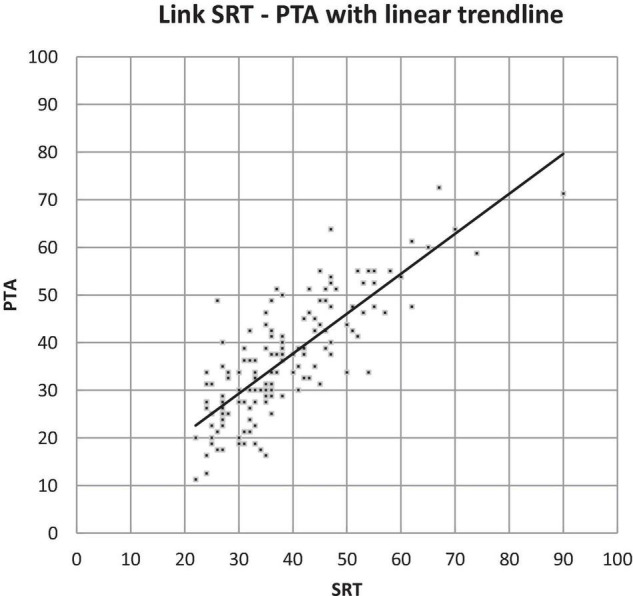
The link between PTA and SRT with a linear trend line.

We compared the results of the pure-tone and speech audiometry (PTA and SRT). This showed that the overall values of SRT were slightly higher than those of PTA; however, when looking at the interclass correlation coefficient, we saw that the PTA and SRT of the better ear had a high correlation of 0.80 (*p* < 0.001) (Figure 5).

### Comparison of BUTTERFLY Data to ISO Standard

**FIGURE 6:**
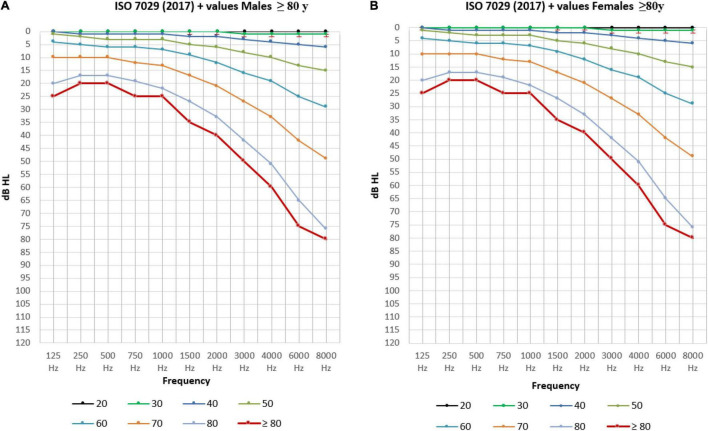
The mean air-conduction thresholds (dB HL) in the better hearing ear for males **(A)** and females **(B)** from 18 to 80 years old according to ISO-values (Technical Committee ISO/TC 43 Acoustics, 2017) completed with the mean air-conduction threshold (dB HL) in the better hearing ear of the elderly ≥ 80 years old in the BUTTERFLY study.

In Figure 6, the ISO-hearing thresholds for different ages from 18 to 80 (P50) are compared to our own data regarding the mean air-conduction threshold in the better hearing ear in our senior population of ≥ 80 years old.

**FIGURE 7:**
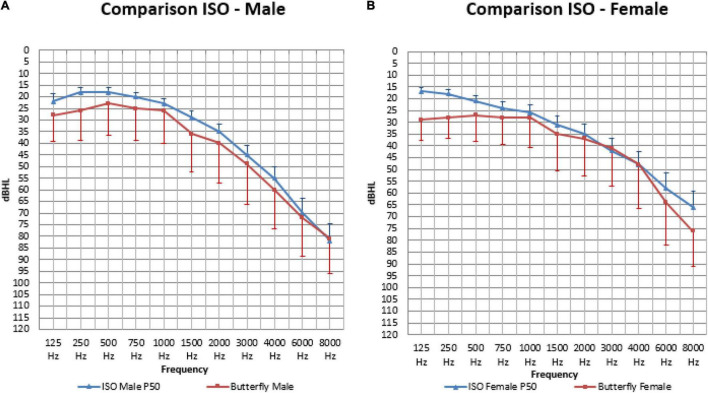
The mean air-conduction thresholds (dB HL) in the better hearing ear for males **(A)** and females **(B)**, compared to the standard ISO median value calculated for every participant based on their sex and age for frequencies from 125 Hz to 8,000 Hz.

In Figure 7, the mean air-conduction threshold in the better hearing ear in our senior population for males and females is compared to the standard ISO median value calculated for every participant based on their sex and age for frequencies from 125 Hz to 8,000 Hz.

When calculating the area under the curve, the measured thresholds were overall significantly higher (or worse) than the calculated ISO thresholds in males (410.98 and 364.78: *p* < 0.001) and females (387.35 and 345.23: *p* = 0.002).

In males (Figure 7A) our mean measured thresholds were significantly worse (or higher) than the calculated ISO thresholds on all frequencies except for 1,000 Hz (25.65 and 22.79 dB HL: *p* = 0.055), 6,000 Hz (72.14 and 69.78 dB HL: *p* = 0.19) and 8,000 Hz (81.48 and 82.28 dB HL: *p* = 0.64). This means that on the following frequencies the ISO calculation was not representative for our population that had worse hearing thresholds: frequency 125 (27.61 and 22.27 dBHL: *p* < 0.01), 250 (25.53 and 18.46 dB HL: *p* < 0.001), 500 (23.15 and 17.83 dB HL: *p* < 0.001), 750 (25.00 and 19.95 dB HL: *p* = 0.001), 1,500 (35.65 and 28.98 dB HL: *p* < 0.001), 2,000 (39.64 and 34.81 dB HL: *p* = 0.008), 3,000 (49.40 and 45.26: *P* = 0.029) and 4,000 Hz (60.23 and 54.60 dB HL: *p* = 0.003).

In females (Figure 7B) our mean measured thresholds were also worse (or higher) than the calculated ISO thresholds but only significant on 125 (28.80 and 16.83 dB HL: *p* < 0.001), 250 (27.61 and 17.72 dB HL: *p* < 0.001), 500 (27.08 and 20.83 dB HL: *p* < 0.001), 750 (27.68 and 23.75 dB HL: *p* = 0.005), 6,000 (63.78 and 57.99 dB HL: *p* = 0.021) and 8,000 Hz (75.74 and 64.78 dB HL: *p* < 0.001).

## Discussion

In our study, hearing loss (> 20 dB) was very common, affecting 90.7% of the senior adults between 80 and 90 years old. In previous studies investigating a population of 80 years and older, hearing loss (≥ 25 dB) was reported in 78.2% (Lin et al., 2011b) to 90.0% of the participants (Cruickshanks et al., 1998). When higher thresholds were used (> 30 dB), hearing loss was reported in 55% of the males and 45% of the females (Roth et al., 2011). Comparing results in different studies was hampered due to the use of different methodologies. The prevalence of hearing loss therefore varied substantially depending on the definitions that were being used: the differences in cut-off thresholds and classification systems for hearing loss, heterogeneity of the measurements and whether these values are calculated for the better or for the worse ear. Since hearing loss is not a clearly defined status, but a gradual condition, the difference between normal and abnormal must be described accurately using definitions and classification systems. The lack of uniformity reduces the potentially comparable data. This demonstrates the need for standardization in the audiometric domain.

Most studies concerning the hearing level of senior adults and ARHL only determine the prevalence of hearing loss and the general trend of hearing thresholds (Lin et al., 2011b; Roth et al., 2011). Frequency distributions by percentile and detailed reference values that are both recent and obtained from actual research participants are more difficult to find, especially in a senior population over 80 years of age (Parving et al., 1997; Hietanen et al., 2004). Most studies are carried out on a larger group of subjects starting from the age of 50 or 60 (Fransen et al., 2008). When we look at the number of participants over 80 years of age it is often a small group compared to the younger participants (Cruickshanks et al., 1998; Roth et al., 2011; Hesse et al., 2014). Various studies have previously concluded that there is a need for further research on this topic as there still are contradictory conclusions about sex difference (Gates and Cooper, 1991; Blanchet et al., 2008). This is possibly due to small sample sizes and the difficulties in obtaining reliable information on the hearing tests in senior adults of 80 years and older (Parving et al., 1997).

In this study we only included subjects who are 80 years and older and focus thereby specifically on the only group not covered by the ISO standards for hearing measurements. However, we have to note that the mean age was 82.4; which means that the largest part of our sample (80%) was situated in age group 1 (80–84).

With the detailed percentile distribution of hearing thresholds in our older age group we hope to contribute to a wider understanding and provide data that can serve as a baseline in order to evaluate the evolution of hearing thresholds among those few people that attain unprecedented ages.

Apart from the prevalence of hearing loss, we determined a representation of the statistical distribution of hearing thresholds on different frequencies related to age and sex. In Figure 6 the ISO-values (Technical Committee ISO/TC 43 Acoustics, 2017) are completed with our own data regarding the mean air-conduction threshold in the better hearing ear in our senior population of ≥ 80 years old. Clearly, there is a further decline in hearing after the age of 80 for both males (Figure 6A) and females (Figure 6B) following the same trend as the ISO-values. However, this further decline is not following the same predicted formula given by ISO. When we calculate the mean ISO thresholds for our participants and compare this to the measured values we see that the ISO-values may be an underestimation of hearing loss in people of 80 years and older. In our population we see that the measured hearing thresholds are significantly worse (or higher) than the calculated ISO thresholds for this same population in males and females when looking at the overall audiogram.

This study demonstrated the deterioration of hearing after the age of 80 years. The deterioration in elderly has been described most pronounced at the middle to higher frequencies above 1,000 Hz (Weinstein, 2002). Our study confirms the known vulnerability in these frequencies. Furthermore, hearing loss has been found to be worse in males than in females, especially at the higher frequencies (Davis, 1989; Cruickshanks et al., 1998; Lin et al., 2011b). Our present study confirmed that males had significantly poorer hearing than females in the high frequencies. In the PTA frequencies this effect was only significant in participants of 85 years and older. On the other hand, in the lower frequencies males had significantly better hearing than females, but only in the younger age group. In the mean audiograms in this study, as is described in literature (Weinstein, 2002), males exhibit a greater decline in the high frequency range. This is clearly demonstrated in the sharply sloping pattern of their pure-tone audiogram. Females exhibit a greater decline in the low frequency range compared to males, which gives them a gradually sloping curve.

Comparing individual hearing tests to age-matched norms gives healthcare providers the opportunity to correctly diagnose people with hearing levels that are below average for their age and sex. Diagnosing and treating hearing loss in senior adults above the age of 80 in a standardized way may prevent the consequences of hearing loss in this vulnerable population. The benefits of early diagnosis and possible treatment will not only affect the hearing capacity of the elderly, but can potentially prevent important outcomes of untreated hearing loss such as social isolation (Mick et al., 2014), depression (Kramer et al., 2002) and cognitive decline (Livingston et al., 2020).

Our sample included 31 hearing aid users. They had a significantly poorer pure-tone audiometry on all frequencies as well as a poorer SRT compared to participants who did not use hearing aids. This means that participants with poorer hearing were more likely to be using a hearing aid (Lin et al., 2011b). This can be explained by the extra motivation to try a treatment to improve daily communication. On the other hand, we see that a large group with hearing loss of more than 40 dB remain undertreated. More than one third (38%) of the participants who could benefit from a hearing aid (hearing loss > 40 dB) didn’t have one. These participants all live in Belgium where a reimbursement is available and hearing centers are easy reachable. Hearing loss is still an underestimated problem in our senior population. It is often misunderstood or even stigmatized. Given the link between hearing loss and cognitive decline, this is a potential modifiable factor where not only the individual but the whole society could benefit from. One third of the hearing aid wearing participants was not satisfied by the effect. This is another undertreated group that potentially could benefit from other forms of hearing advancements such as cochlear implantation.

It is widely accepted that the prevalence of hearing loss is higher with increasing age. In our study we observed that the older age group indeed had a significantly poorer hearing than the younger group, and that males were more affected by age than females on all frequencies.

We noticed increasing difficulties in speech recognition in our older population. It has been described that speech audiometry is significantly better in females compared to males (Blanchet et al., 2008). In our study this difference was only clear in the older age group. This can be explained by the fact that results in males significantly deteriorated with age, whereas those in females did not.

When comparing the results of the pure-tone and speech audiometry in our study we saw a high correlation between SRT and PTA, yet the overall distribution showed that the SRT was slightly higher than the PTA, or the recognition of speech was poorer than the detection of pure tones. We hypothesize that when the SRT is higher (or worse) than the PTA, there is a higher possibility of central processing problems or central presbycusis.

Our audiometric data was used to investigate the link between hearing loss, hearing aid usage and cognitive decline (Vella Azzopardi et al., 2021). This study showed an association between hearing loss and global and domain-specific (processing speed, selective and alternating attention) cognitive decline in male non-hearing aid users.

Our study has some limitations. The first is that information on the history of the participants was gathered by using a questionnaire, filled out by the participants together with the ENT specialist. All the information gathered is thus based on memory, which may not be sufficient when answering such specific questions. A recall bias must be considered, for example, while military service had been obligatory for males of that generation, only 12 of the 84 males mentioned noise exposure related to military practice. Participants could have been exposed to noise or ototoxic drugs without knowing. This is why noise exposure was not used as an exclusion criterion. This has to be considered when comparing our data to other studies where the noise-exposed participants were excluded. The interpretation of the answers by different ENT specialists while completing the questionnaire could have an influence on the standardization. Also, in our study we only have 9 participants who complained of tinnitus, although tinnitus is often linked with high frequency losses (Shargorodsky et al., 2010), which are very frequent in our population. It is possible that the lack of systematically asking about the presence of tinnitus has caused an underestimation of the tinnitus complaints in our study sample. The inequality in the numbers of participants in age group 1 and 2 and therefore the low numbers in age group 2 should be noted. With a mean age of 82.4, our sample stays in the range of the already existing ISO values, however, when we can observe a difference in hearing thresholds even with this minor extension in age, this emphasizes the need for further research in this domain.

Another limitation is the fact that audiometric analysis was only performed by pure-tone and speech audiometry. Other tests like the speech perception in noise (SPIN) test could have provided more detailed results, however, the participants underwent multiple tests across hospital services in 1 day and additional tests could have led to negative effects of fatigue (McGarrigle et al., 2014) and reduced attention.

A third limitation is that our population was not randomly selected but had to meet the following inclusion criteria: walking independently and living at home independently. Consequently, this is not a representation of the entire 80 + -population and may lead to a bias in hearing measurements. It has been described in literature that hearing loss has an influence on functional decline (Gopinath et al., 2012). People with more severe hearing loss are therefore more likely to not fit the initial inclusion criteria. As a result, our values could represent an underestimation of hearing loss in senior adults.

To conclude, it should be noted that, when comparing the calculated ISO-values to our measured thresholds, these measurements were done in audiometric setting where all numbers are a multiple of 5. When calculating a mean threshold by using the ISO formula all numbers are possible.

## Conclusion

Hearing loss is a very common problem in people over 80 years of age. Our study demonstrates that a large majority (90.7%) of the senior adults suffer from hearing loss greater than 20 dB HL. In our population, males’ hearing sensitivity tends to decline more with increasing age on all frequencies with a steeply downward-sloping curve. This in contrast to females, who’s hearing sensitivity declines with a more gradually sloping curve. Our findings suggest that the ISO-values may be an underestimation of hearing loss in people of 80 years and older both in males and females. Therefore, we present a statistical distribution of hearing thresholds on different frequencies related to age and sex that can be used as a baseline for further development of reference values for this older age group. A large part of the older age group with hearing loss stay undertreated. More than one third (38%) of the participants who could benefit from hearing aid (hearing loss > 40 dB) didn’t have one, and one third of the hearing aid wearing participants was not satisfied by the effect and could potentially benefit from other hearing advancement such as cochlear implantation.

## Data Availability Statement

The original contributions presented in this study are included in the article. Further inquiries can be directed to the corresponding author.

## Ethics Statement

The studies involving human participants were reviewed and approved by Commissie voor Medische Ethiek Universitair Ziekenhuis Brussel. The patients/participants provided their written informed consent to participate in this study.

## Author Contributions

KDR conceptualized and designed this study, analyzed data, drafted the initial manuscript, and approved the final manuscript as submitted. RVA recruited the patients and did the general medical evaluation. EL performed the audiometric testing’s at UZ Brussel, reviewed and helped with the acquisition of data. KDR and RB provided statistical analysis. IBe and IBa designed and supervised the BUTTERFLY experiment. IF, OM, VT, and FG provided critical revision. All authors reviewed and approved the final manuscript as submitted.

## Conflict of Interest

The authors declare that the research was conducted in the absence of any commercial or financial relationships that could be construed as a potential conflict of interest.

## Publisher’s Note

All claims expressed in this article are solely those of the authors and do not necessarily represent those of their affiliated organizations, or those of the publisher, the editors and the reviewers. Any product that may be evaluated in this article, or claim that may be made by its manufacturer, is not guaranteed or endorsed by the publisher.
